# CT-based radiomics deep learning signatures for non-invasive prediction of metastatic potential in pheochromocytoma and paraganglioma: a multicohort study

**DOI:** 10.1186/s13244-025-01952-4

**Published:** 2025-04-05

**Authors:** Yongjie Zhou, Yuan Zhan, Jinhong Zhao, Linhua Zhong, Fei Zou, Xuechao Zhu, Qiao Zeng, Jiayu Nan, Lianggeng Gong, Yongming Tan, Lan Liu

**Affiliations:** 1 Department of Radiology, Jiangxi Cancer Hospital & Institute, Jiangxi Clinical Research Center for Cancer, The Second Affiliated Hospital of Nanchang Medical College, Nanchang, China; 2 Department of Pathology and Institute of Molecular Pathology, The First Affiliated Hospital, Jiangxi Medical College, Nanchang University, Nanchang, China; 3 Department of Radiology, The Second Affiliated Hospital, Jiangxi Medical College, Nanchang University, Nanchang, China; 4 Department of Radiology, The First Affiliated Hospital, Jiangxi Medical College, Nanchang University, Nanchang, China

**Keywords:** Pheochromocytoma, Paraganglioma, Metastatic potential, Radiomics, Deep learning

## Abstract

**Objectives:**

This study aimed to develop and validate CT-based radiomics deep learning signatures for the non-invasive prediction of metastatic potential in pheochromocytomas and paragangliomas (PPGLs).

**Methods:**

We conducted a retrospective analysis of 249 PPGL patients from three institutions, dividing them into training (*n* = 138), test1 (*n* = 71), and test2 (*n* = 40) sets. Based on the grading system for adrenal pheochromocytoma and paraganglioma (GAPP), patients were classified into low-risk (GAPP < 3) and high-risk (GAPP ≥ 3) groups. Radiomic features were extracted from CT venous phase images and modeled using six machine learning algorithms. The maximum 2D sections and 3D images of each tumor were input into four ResNet models to obtain predictive probabilities. Optimal models were selected based on receiver operating characteristic analysis and integrated with radiological features to develop a combined model, which was evaluated on external datasets, and explored prognostic information.

**Results:**

The support vector machine radiomics and 2D ResNet-50 models demonstrated good performance. By integrating these two models with intratumoral necrosis features, we constructed a combined model that achieved high accuracy, with area under the curve (AUC) values of 0.90 for the training, 0.86 for the test1, and 0.88 for the test2 sets. This model effectively stratified patients based on metastasis-free survival (*p* = 0.003). Its predictive ability remains robust below the 6 cm threshold, with AUC values exceeding 0.87 across all datasets.

**Conclusions:**

The combined model can predict the metastatic potential of PPGL in the preoperative stage, providing a precise surgical strategy for pheochromocytoma regarding the 6 cm surgical threshold.

**Critical relevance statement:**

The combined model, established based on radiomic and deep learning signatures, shows potential for early preoperative prediction of metastatic potential in PPGL.

**Key Points:**

- Metastatic potential of PPGL affects surgical approaches and prognosis.
- CT-based radiomics deep learning signatures can predict the metastatic potential in PPGL.3. The combined model’s predictive ability remains robust below the 6-cm threshold.
- The combined model’s predictive ability remains robust below the 6-cm threshold.

**Graphical Abstract:**

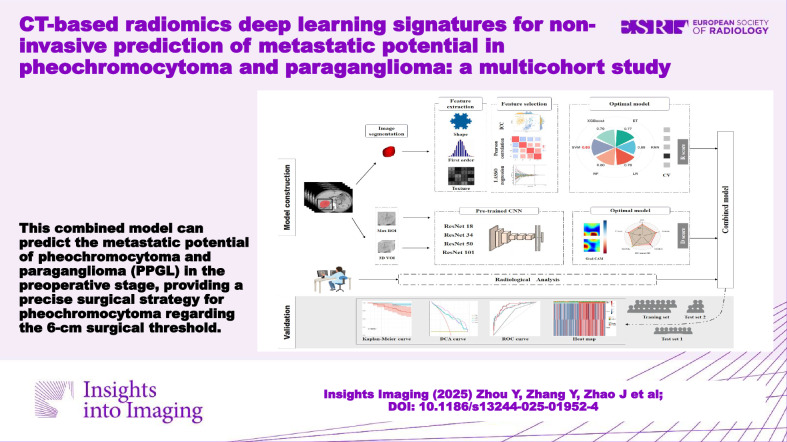

## Introduction

Pheochromocytomas and paragangliomas (PPGLs) are rare types of neuroendocrine tumors characterized by the secretion of catecholamines. PPGLs encompass pheochromocytomas from the adrenal medulla and paragangliomas that originate outside the adrenal gland [1]. With the rapid advancements in medical imaging technology, the diagnostic rate of PPGLs has increased. The World Health Organization updated this classification in 2017, replacing the term “malignant PPGLs” with “metastatic PPGLs” [2]. Currently, studies on large patient cohorts report that about 20% of PPGLs develop metastases [3, 4]. The median survival of patients with metastatic PPGLs is 6.7 years [5], and the 5-year and 10-year mortality rates are 37% (from 7 studies with *n* = 738) and 29% (from 2 studies with *n* = 55), respectively [6]. Surgery remains the primary treatment option for PPGL. The size of the tumor is a significant risk factor for the metastasis of pheochromocytoma [3, 7, 8]. Endocrine guidelines and consensus indicate that for patients with pheochromocytomas larger than 6 cm, or those with metastasis, open resection is preferred over laparoscopic surgery to ensure complete tumor removal and to avoid local recurrence or implantation metastasis [9–11]. However, it is important to note that tumors smaller than 6 cm may also exhibit the potential for distant metastasis, which can shorten the patient’s disease-related survival [12, 13]. Therefore, the early identification of patients with metastatic potential is essential for determining which individuals would benefit from laparoscopic surgery.

In 2014, the Japanese pheochromocytoma research group proposed the grading system for adrenal pheochromocytoma and paraganglioma (GAPP) [14]. This system assigns scores based on histological characteristics, such as growth pattern, cell count, microvascular infiltration, and the Ki67 labeling index, to categorize PPGLs into well-differentiated (0–2 points), moderately differentiated (3–6 points), and poorly differentiated (7–10 points). The likelihood of metastasis is significantly lower in well-differentiated cases (GAPP < 3) compared to those that are moderately or poorly differentiated (GAPP ≥ 3). Multiple studies indicate that this grading system is closely related to the metastatic risk and survival prognosis of PPGLs [15–19]. Currently, the evaluation of GAPP relies on organ pathology and immunohistochemical assessments, which are complex and costly [14]. Thus, it is essential to develop a simpler, non-invasive method for predicting the GAPP of PPGLs.

Computed tomography (CT) is the imaging technique of choice for PPGLs, serving an essential role in the preoperative diagnosis and assessment of these tumors [1]. However, radiologists still face challenges in assessing the metastatic potential of PPGLs using conventional CT images. Radiomics, a quantitative medical imaging technique that utilizes high-throughput extraction of tumor region features [20, 21], has successfully predicted tumor differentiation and risk stratification for various malignancies [22–24]. In recent years, deep learning (DL) technologies have emerged as a new direction in radiomics research, allowing for the autonomous extraction of quantitative representations from medical images [25, 26]. Among these technologies, convolutional neural networks (CNNs) have become the most widely utilized DL model [27]. Additionally, deep transfer learning enables the application of DL to small datasets by fine-tuning pre-trained DL networks for new tasks [28]. Both radiomics and DL technologies are evolving rapidly, with radiomics offering high interpretability in the analysis of high-throughput imaging data. Currently, there is no authoritative declaration or consensus suggesting that DL outperforms radiomics. Thus, combining DL with radiomic features may enhance the predictive capability for GAPP. While recent studies have demonstrated the feasibility of predicting GAPP based on radiomic features [29], the integrative approach of employing both radiomics and DL for the non-invasive prediction of metastatic potential in PPGLs has not been adequately explored in the literature.

This multicenter study aims to develop and validate a radiomic DL signature based on enhanced CT imaging for the non-invasive identification of metastatic potential in patients with PPGL.

## Methods

### Participants

**Fig. 1:**
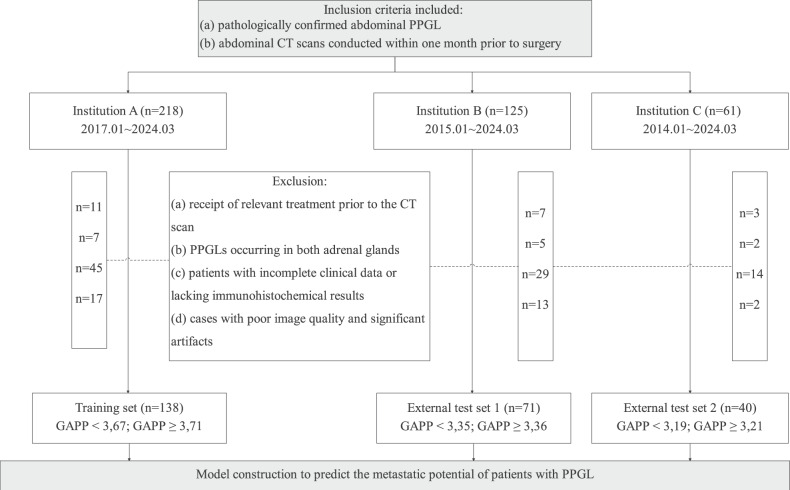
Flowchart illustrating the inclusion and exclusion criteria for this study. CT, computed tomography; PPGL, pheochromocytoma and paraganglioma

This retrospective study was approved by the ethics committees of the First Affiliated Hospital of Nanchang University (Institution A), the Second Affiliated Hospital of Nanchang University (Institution B), and the Jiangxi Cancer Hospital (Institution C), waiving the need for written informed consent. The dataset includes 404 patients diagnosed with abdominal PPGLs at these institutions from January 2014 to March 2024. The inclusion criteria for all patients were as follows: (a) pathologically confirmed abdominal PPGL; (b) abdominal CT scans conducted within one month prior to surgery, including both non-contrast and contrast-enhanced scans. The exclusion criteria are as follows: (a) receipt of relevant treatment prior to the CT scan; (b) PPGLs occurring in both adrenal glands, which could affect the evaluation of catecholamine types in the GAPP score; (c) patients with incomplete clinical data or lacking immunohistochemical results; (d) cases with poor image quality and significant artifacts. The inclusion and exclusion process is detailed in Fig. 1. Ultimately, 249 patients were included in the analysis, comprising 138 from Institution A as the training set, 71 from Institution B as the external test set 1, and 40 from Institution C as the external test set 2.

It is noteworthy that 201 patients have been previously described [29]. Whereas the prior work aimed at risk stratification based on multiphasic CT radiomics, this study focused on the use of venous phase imaging radiomics DL signatures for the non-invasive prediction of metastatic potential in PPGL, supplemented by prognostic analysis.

### CT imaging acquisition

This study utilized various CT scanners from three institutions. Each patient underwent a triphasic CT scan, which included non-contrast, arterial, and venous phases. Detailed information regarding the specific scanning configurations and settings of the CT equipment can be found in Supplementary Table S1.

### Clinical data and radiological features

**Fig. 2:**
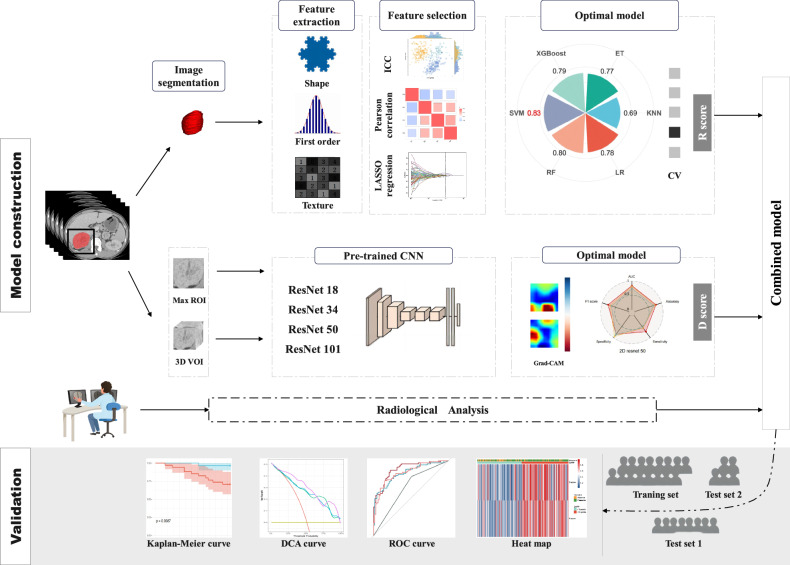
Workflow diagram. ICC, intra-class correlation coefficient; LASSO, least absolute shrinkage and selection operator; CV, cross-validation; R score, radiomics signature score; D score, deep learning signature score; ROC, receiver operating characteristic; DCA, decision curve analysis

Clinical data for each patient were recorded, including age, gender, hypertension history, and catecholamine levels prior to surgery. Two experienced radiologists (Reader 1 with 9 years and Reader 2 with 12 years in abdominal diagnostics) independently assessed the CT characteristics of the cases, which they identified as PPGLs without knowing the histopathology. A third radiologist with 25 years of experience will resolve any discrepancies. The evaluated CT characteristics included: (a) maximum tumor diameter (MTD); (b) tumor location; (c) intratumoral calcification; (d) intratumoral hemorrhage; (e) tumor margins; (f) intratumoral necrosis; and (g) intratumoral vascular penetration (IVP). Detailed descriptions of the radiological characteristics can be found in the Supplementary Materials, while the overall workflow is illustrated in Fig. 2.

### Pathological assessment and follow-up

Histological slides from three medical institutions were assessed by two pathologists with 9 years of experience, without knowledge of clinical data, following standardized analysis protocols. In cases of discrepancies in the assessment results, a third senior pathologist was appointed to resolve the disagreement. Cohen’s kappa coefficient was employed to assess the reliability among pathologists. According to the GAPP criteria, the metastatic potential of PPGLs is categorized into a low-risk group (GAPP < 3) and a high-risk group (GAPP ≥ 3). Detailed information and results regarding the GAPP criteria can be found in Supplemental Tables S2 and S3.

Patient prognostic information was obtained through the electronic medical record system and via telephone follow-up. To ensure sufficient follow-up time, cases from Institution A (January 2017 to March 2019) and Institution C (January 2014 to March 2019) were included. The duration of metastasis-free survival (MFS) is defined as the time from the date of surgery to either the date of tumor metastasis or the last follow-up date, March 2024.

### Image preprocessing, radiomics feature extraction, and selection

To resolve inconsistencies in raw CT images caused by varying scanning devices across hospitals, we preprocessed all images. We standardized the voxel size to 1 × 1 × 1 mm³ and adjusted the window level and width to 0 Hu and 400 Hu, respectively, to enhance the segmentation of the region of interest (ROI). Additionally, we set the bin width to 25 for image discretization.

In the absence of patients’ histopathological results, a radiologist (Reader 1) manually delineated the tumor margins on the venous phase images from the enhanced scan using ITK-SNAP software (version 3.6.0, http://www.itksnap) [30]. During the delineation process, we ensured that adjacent normal tissue and blood vessels were excluded, while referencing reconstructed coronal and sagittal images to clarify the spatial relationship surrounding the lesions. This process resulted in a comprehensive three-dimensional volume of the tumor for subsequent feature extraction. We randomly selected 30 patients (17 from the low-risk group and 13 from the high-risk group). Reader 1 conducted two assessments separated by one month, referred to as ROI1 and ROI2, while Reader 2 performed a single assessment, termed ROI3. Intraclass correlation coefficients (ICCs) were used to evaluate intra- and interobserver agreement in feature extraction, with an ICC value ≥ 0.75 indicating strong reliability of the measurements.

Feature extraction was performed using the “PyRadiomics” software package [31], following the guidelines of the Imaging Biomarker Standardization Initiative [21]. From the venous phase images, we extracted 1106 radiomic features from each tumor’s three-dimensional volume, including shape, first-order, and texture features such as the gray-level co-occurrence matrix, gray-level size zone matrix, gray-level run-length matrix, neighboring gray-tone difference matrix, gray-level dependence matrix, and wavelet transformation features. Due to significant redundancy among these extracted radiomic features, we first standardized all features and then calculated the Pearson correlation coefficients between them. If any two features had a correlation coefficient exceeding 0.9, we retained only one to reduce redundancy. Finally, to maximize the descriptive power of the features, we employed the least absolute shrinkage and selection operator (LASSO) regression model for final feature selection. By adjusting the penalty parameter λ, we were able to shrink the coefficients of irrelevant features to zero. Ultimately, we used 10-fold cross-validation to identify the optimal λ and selected the final features based on the non-zero coefficients.

### Development of radiomics and DL models

We selected six mainstream machine learning algorithms for model construction using the final filtered radiomic features, including logistic regression (LR), support vector machine (SVM), k-nearest neighbors (KNN), random forest (RF), extra trees (ET), and eXtreme gradient boosting (XGBoost) [32]. We performed 5-fold cross-validation and grid search for parameter optimization on the training set, and validated model performance on external datasets. The receiver operating characteristic (ROC) curve was employed to compare the performances of the models, enabling us to select the optimal radiomic model. Based on its predicted probabilities, we defined the R score (radiomic signature score).

The CNN-based ResNet model is not significantly affected by the vanishing gradient problem, allowing it to effectively handle complex medical image data [33]. Therefore, this study selected the ResNet model to analyze the venous phase images of 249 patients with PPGL. To mitigate the limitations posed by the small sample size, we implemented transfer learning utilizing a pre-trained ImageNet model (http://www.image-net.org). During training, we used 2D images based on the maximal ROI and 3D images encompassing the entire tumor as input. In this study, ResNet18, ResNet34, ResNet50, and ResNet101 were employed. Detailed parameter settings for these models are available in the supplementary materials. We obtained predictive probabilities for each CNN model from the validation and test sets, followed by performance comparison using ROC curves. Ultimately, we selected the optimal DL model, utilizing its predictive probabilities to define the D score (deep learning signature score).

### Validation and construction of the combined model

We sequentially conducted univariate and multivariate LR analyses to integrate the R score, D score, and radiological features in order to construct a combined model. The results of the model were visualized using a heat map, and the performance of each model was evaluated using various metrics, including the ROC curve and its area under the curve (AUC), as well as accuracy, specificity, sensitivity, and F1 score. Furthermore, calibration curves and decision curve analysis (DCA) were conducted to assess the consistency of the model predictions and their clinical net benefit. Additionally, we employed MFS-based Kaplan–Meier analysis to evaluate the predictive capabilities of the GAPP and combined models regarding the metastatic potential of PPGLs, using the median as the cutoff value, and we conducted a log-rank test to analyze intergroup differences.

### Statistical analysis

The content of statistical analysis is detailed in the Supplementary Materials.

## Results

### Baseline characteristics of the patients

A total of 249 patients were included in this study, comprising 128 males (mean age, 50.5 ± 12.7 years) and 121 females (mean age, 49.5 ± 14 years). The distribution of clinical radiological features was similar across the training set (*n* = 138), external test set 1 (*n* = 71), and test set 2 (*n* = 40), with all *p*-values exceeding 0.05. Based on the GAPP criteria, in the training set, 67 patients (48.6%) were classified as a low-risk group, while 71 patients (51.4%) were categorized as a high-risk group. In the test set 1, 35 patients (49.3%) were classified as low-risk group, and 36 patients (50.7%) were classified as high-risk group. In the test set 2, 19 patients (47.5%) were categorized as low-risk group, and 21 patients (52.5%) were classified as high-risk group. Detailed clinical information can be found in Table 1.

**Table 1:** Clinical and radiological characteristics of PPGL patients in different datasets

Characteristic	Dataset, No. (%)				*p*-value
	Total sets (*n* = 249)	Training set (*n* = 138)	External test set 1 (*n* = 71)	External test set 2 (*n* = 40)	
Age (years)					
Median [IQR]	51 [43, 60]	51 [44, 59]	51 [43, 61]	53 [43, 59]	0.976
Sex					
Female	121 (48.594)	65 (47.101)	34 (47.887)	22 (55.000)	0.672
Male	128 (51.406)	73 (52.899)	37 (52.113)	18 (45.000)	
Hypertension					
No	150 (60.241)	90 (65.217)	42 (59.155)	18 (45.000)	0.069
Yes	99 (39.759)	48 (34.783)	29 (40.845)	22 (55.000)	
Location					
Adrenal	198 (79.518)	108 (78.261)	57 (80.282)	33 (82.500)	0.828
Extra-adrenal	51 (20.482)	30 (21.739)	14 (19.718)	7 (17.500)	
MTD					
≤ 6 cm	188 (75.502)	99 (71.739)	57 (80.282)	32 (80.000)	0.306
> 6 cm	61 (24.498)	39 (28.261)	14 (19.718)	8 (20.000)	
Hemorrhage					
Absence	206 (82.731)	115 (83.333)	57 (80.282)	34 (85.000)	0.788
Presence	43 (17.269)	23 (16.667)	14 (19.718)	6 (15.000)	
Calcification					
Absence	224 (89.960)	122 (88.406)	66 (92.958)	36 (90.000)	0.584
Presence	25 (10.040)	16 (11.594)	5 (7.042)	4 (10.000)	
Margin					
Clear	175 (70.281)	99 (71.739)	47 (66.197)	29 (72.500)	0.67
Unclear	74 (29.719)	39 (28.261)	24 (33.803)	11 (27.500)	
IVP					
Absence	138 (55.422)	83 (60.145)	37 (52.113)	18 (45.000)	0.19
Presence	111 (44.578)	55 (39.855)	34 (47.887)	22 (55.000)	
Necrosis					
Absence	79 (31.727)	48 (34.783)	20 (28.169)	11 (27.500)	0.512
Presence	170 (68.273)	90 (65.217)	51 (71.831)	29 (72.500)	
GAPP					
Low-risk	121 (48.594)	67 (48.551)	35 (49.296)	19 (47.500)	0.984
High-risk	128 (51.406)	71 (51.449)	36 (50.704)	21 (52.500)	

*MTD* maximum tumor diameter, *IVP* intratumoral vascular penetration, *GAPP* grading system for adrenal PPGLs

### Validation and construction of the radiomic model

In this study, a total of 1081 radiomic features had ICC values ≥ 0.75. After filtering, we retained six non-zero coefficient features, including two original shape features, two first-order features, and two texture features (Fig. S1).

**Fig. 3:**
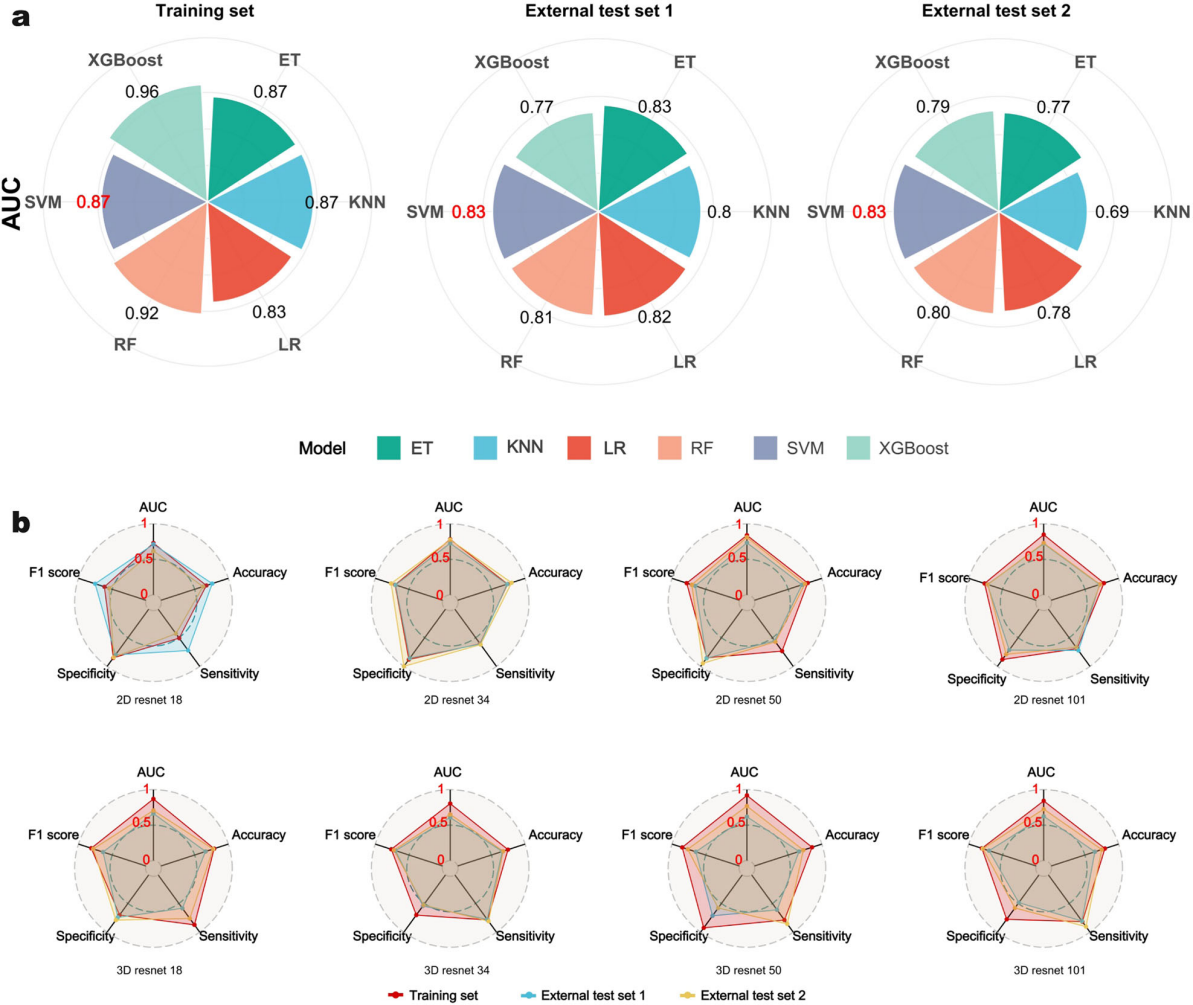
**a** Petal chart visualization of the predictive performance of different machine learning models. **b** Radar chart visualization of the predictive performance of various DL models

We combined the final selected six radiomic features into six machine learning algorithms: LR, SVM, KNN, RF, ET, and XGBoost, to create models and compare their AUC values. As shown in Fig. 3a and Table 2, the XGBoost model achieved the highest AUC value of 0.96 in the training set. However, its performance on the external test sets was inferior to that of the SVM model. Therefore, we selected the SVM model to establish the radiomics model. The AUC values for external test set 1 and test set 2 are both 0.83, exceeding those of other machine learning models. This indicates a high predictive diagnostic value. The probability predictions based on the SVM model are defined as the R score, which represents the radiomic signature score.

**Table 2:** The predictive performance of various machine learning and DL models

Model	AUC (95% CI)	Accuracy	Sensitivity	Specificity	F1 score
Training set					
LR	0.83 (0.76–0.89)	0.76	0.73	0.79	0.76
SVM^*^	0.87 (0.81–0.93)	0.78	0.69	0.87	0.76
KNN	0.87 (0.81–0.92)	0.75	0.61	0.91	0.72
RF	0.92 (0.88–0.96)	0.84	0.72	0.97	0.82
ET	0.87 (0.81–0.92)	0.78	0.63	0.94	0.75
XGBoost	0.96 (0.94–0.99)	0.89	0.85	0.94	0.89
2D ResNet18	0.73 (0.66–0.81)	0.67	0.51	0.85	0.62
2D ResNet34	0.78 (0.69–0.86)	0.74	0.61	0.88	0.70
2D ResNet50^*^	0.84 (0.78–0.90)	0.79	0.73	0.85	0.78
2D ResNet101	0.85 (0.80–0.91)	0.78	0.69	0.88	0.77
3D ResNet18	0.87 (0.81–0.92)	0.79	0.87	0.70	0.81
3D ResNet34	0.80 (0.74–0.86)	0.75	0.79	0.70	0.76
3D ResNet50	0.92 (0.88–0.96)	0.86	0.79	0.93	0.85
3D ResNet101	0.84 (0.77–0.91)	0.80	0.82	0.78	0.81
External test set 1					
LR	0.82 (0.72–0.92)	0.79	0.75	0.83	0.78
SVM^*^	0.83 (0.72–0.93)	0.79	0.75	0.83	0.78
KNN	0.80 (0.69–0.91)	0.70	0.50	0.91	0.63
RF	0.81 (0.71–0.92)	0.78	0.78	0.77	0.78
ET	0.83 (0.73–0.93)	0.78	0.78	0.77	0.78
XGBoost	0.77 (0.67–0.88)	0.72	0.78	0.66	0.74
2D ResNet18	0.71 (0.60–0.85)	0.76	0.72	0.80	0.75
2D ResNet34	0.73 (0.59–0.84)	0.73	0.61	0.86	0.70
2D ResNet50^*^	0.74 (0.64–0.85)	0.70	0.56	0.86	0.66
2D ResNet101	0.72 (0.58–0.84)	0.72	0.72	0.71	0.72
3D ResNet18	0.66 (0.56–0.77)	0.66	0.58	0.74	0.64
3D ResNet34	0.61 (0.48–0.76)	0.66	0.78	0.54	0.70
3D ResNet50	0.62 (0.47–0.71)	0.66	0.61	0.71	0.65
3D ResNet101	0.63 (0.47–0.74)	0.65	0.81	0.49	0.70
External test set 2					
LR	0.78 (0.63–0.93)	0.78	0.76	0.79	0.78
SVM^*^	0.83 (0.68–0.97)	0.83	0.81	0.84	0.83
KNN	0.69 (0.53–0.85)	0.63	0.38	0.90	0.52
RF	0.80 (0.66–0.94)	0.73	0.81	0.63	0.76
ET	0.77 (0.62–0.93)	0.75	0.86	0.63	0.78
XGBoost	0.79 (0.65–0.93)	0.73	0.76	0.68	0.74
2D ResNet18	0.62 (0.42–0.80)	0.63	0.43	0.84	0.55
2D ResNet34	0.78 (0.63–0.93)	0.80	0.62	1.00	0.76
2D ResNet50^*^	0.81 (0.69–0.95)	0.75	0.57	0.95	0.71
2D ResNet101	0.73 (0.55–0.88)	0.73	0.67	0.79	0.72
3D ResNet18	0.71 (0.56–0.84)	0.78	0.76	0.79	0.78
3D ResNet34	0.65 (0.49–0.82)	0.68	0.81	0.53	0.72
3D ResNet50	0.77 (0.59–0.88)	0.73	0.86	0.58	0.77
3D ResNet101	0.73 (0.54–0.86)	0.75	0.90	0.58	0.79

*LR* logistic regression, *SVM* support vector machine, *KNN* k-nearest neighbors, *RF* random forest, *ET* extra trees, *XGBoost* eXtreme gradient boosting

^*^ Representing the optimal radiomics and DL models

### Validation and construction of the DL model

According to the AUC results for all DL models presented in Fig. 3b and Table 2. These results indicate that the 2D ResNet50 model demonstrated the best discriminative performance, with AUC values of 0.74 on the test set 1 and 0.81 on the test set 2. Consequently, we ultimately chose the predicted probabilities from the 2D ResNet50 model as the D score, defined as the D score. Figure S2 presents examples of Gradient-weighted Class Activation Mapping outputs generated from 2D maximum cross-section images trained using the ResNet model.

### Validation and construction of the combined model

**Fig. 4:**
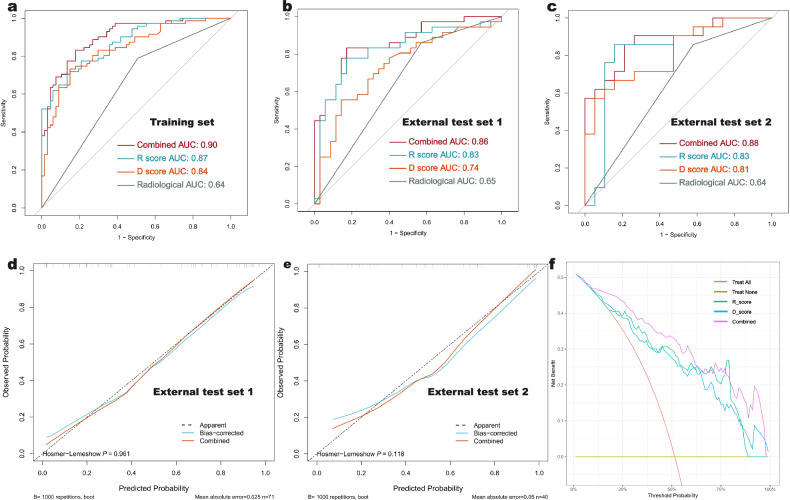
In all datasets, the performance of four models predicting the metastatic potential of PPGL was assessed. The receiver operating characteristic curves for the training set (**a**), external test set 1 (**b**), and external test set 2 (**c**) indicated that the combined model, which integrates radiological features (intratumoral necrosis), R score, and D score, achieved the highest AUC values among all models, with AUCs of 0.90, 0.86, and 0.88, respectively. The radiological model is constructed using the characteristics of intratumoral necrosis. The calibration curves for the combined model in the external test set 1 (**d**) and test set 2 (**e**) are presented. DCA highlights the clinical utility of the combined model, demonstrating a wider range of threshold probabilities compared to the R score and D score models (**f**)

The results of both univariate and multivariate LR analyses indicate that intratumoral necrosis, R score, and D score are independent factors predicting the metastatic potential of PPGLs (Table 3). We developed a combined model based on these three independent predictive factors, which showed an AUC of 0.90 (0.86–0.95) in the training set. In two external test sets, the model achieved AUC values of 0.86 (95% CI: 0.78–0.93) and 0.88 (95% CI: 0.76–0.97), respectively., all were superior to the three individual models (Table 4 and Fig. 4a–c). The calibration curve for the combined model shows a strong consistency between predicted probabilities and actual outcomes (Fig. 4d, e). Additionally, DCA highlights the clinical utility of the combined model, which demonstrates a wider range of threshold probabilities compared to radiomics and DL models (Fig. 4f).

**Table 3:** Perform univariate and multivariate LR analysis in the training set

Characteristic	Univariate analysis		Multivariate analysis	
	OR (95% CI)	*p*-value	OR (95% CI)	*p*-value
Age (mean ± SD)	1.00 (0.98–1.03)	0.815		
Sex (female vs male)	1.49 (0.76–2.93)	0.241		
Hypertension (no vs yes)	0.91 (0.45–1.84)	0.804		
Location (Pheo vs PGL)	1.31 (0.58–2.95)	0.519		
MTD (≤ 6 vs > 6 cm)	7.03 (2.83–17.50)	< 0.001	0.47 (0.10–2.16)	0.332
Hemorrhage (absent vs present)	2.49 (0.95–6.52)	0.062		
Calcification (absent vs present)	4.78 (1.30–17.63)	0.019	2.19 (0.33–14.44)	0.414
Margin (clear vs unclear)	3.94 (1.73–8.95)	0.001	1.07 (0.33–3.48)	0.906
IVP (absent vs present)	2.60 (1.28–5.27)	0.008	0.94 (0.31–2.84)	0.916
Necrosis (absent vs present)	3.62 (1.72–7.63)	< 0.001	3.98 (1.34–11.85)	**0.013**
D_score	316.10 (48.81–2047.12)	< 0.001	51.02 (5.10–510.74)	**<** **0.001**
R_score	664.76 (92.25–4790.32)	< 0.001	409.70 (20.97–8002.68)	**<** **0.001**

*R score* radiomics signature score, *D score* deep learning signature score

Bold values represent *p*-values < 0.05, which are ultimately used to construct the model

**Table 4:** The predictive performance of radiological, R score, D score, and the combined model.

Model	AUC (95% CI)	Accuracy	Sensitivity	Specificity	F1 score
Training set					
Radiological	0.64 (0.59–0.69)	0.64	0.79	0.49	0.70
R score	0.87 (0.81–0.93)	0.78	0.69	0.87	0.76
D Score	0.84 (0.78–0.90)	0.79	0.73	0.85	0.78
Combined	0.90 (0.86–0.95)	0.82	0.83	0.81	0.83
External test set 1					
Radiological	0.65 (0.53–0.75)	0.65	0.86	0.43	0.71
R score	0.83 (0.72–0.93)	0.79	0.75	0.83	0.78
D Score	0.74 (0.64–0.85)	0.70	0.56	0.86	0.66
Combined	0.86 (0.78–0.93)	0.82	0.81	0.83	0.82
External test set 2					
Radiological	0.64 (0.51–0.77)	0.65	0.86	0.42	0.72
R score	0.83 (0.68–0.97)	0.83	0.81	0.84	0.83
D Score	0.81 (0.69–0.95)	0.75	0.57	0.95	0.71
Combined	0.88 (0.76–0.97)	0.83	0.86	0.79	0.84

The radiological model is constructed using the characteristics of intratumoral necrosis

*R score* radiomics signature score, *D score* deep learning signature score

**Fig. 5:**
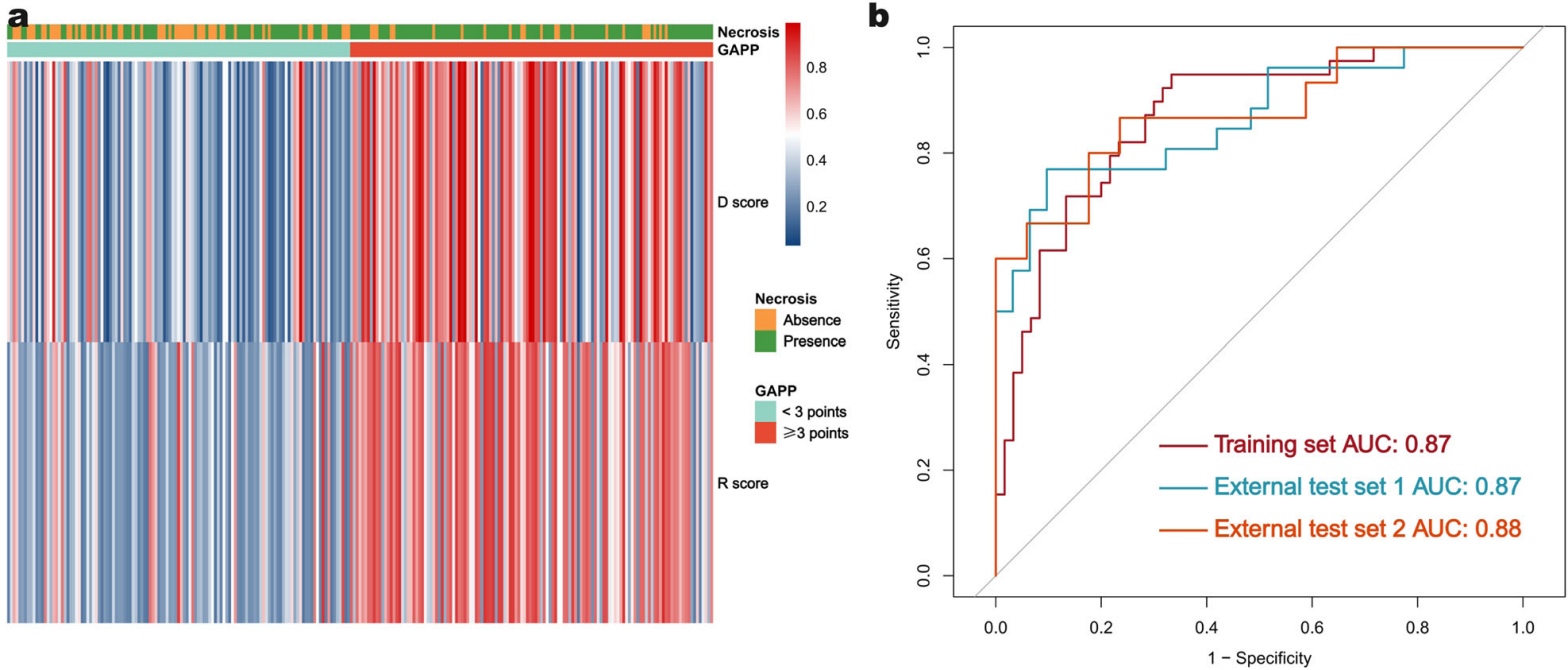
The heatmap (**a**) illustrates the correlation between the metastatic potential of PPGL (according to GAPP criteria) and radiological characteristics (intratumoral necrosis), as well as the R score (radiomic signature score) and D score. Tumors exhibiting intratumoral necrosis, along with higher R scores and D scores, demonstrate a greater metastatic potential. The color bar represents the continuous values of the R scores and D scores, with a minimum value of 0 and a maximum value of 1. **b** Predictive ability for the metastatic potential of pheochromocytomas with a maximum diameter of less than 6 cm, as evaluated in the training set and external test sets 1 and 2

The heat map indicates that PPGL patients with intratumoral necrosis, high R scores, and high D scores are more likely to exhibit metastatic potential (Fig. 5a). To assess the recommendations regarding surgical approaches for pheochromocytoma based on the 6 cm threshold outlined in the endocrine guidelines, we evaluated the predictive capacity of this threshold for metastatic potential. Our findings reveal that for tumors measuring ≤ 6 cm, the predictive performance is outstanding across the training set, test set 1, and test set 2, with AUC values of 0.87 (95% CI: 0.80–0.94), 0.87 (95% CI: 0.77–0.96), and 0.88 (95% CI: 0.75–1.00), respectively (Fig. 5b).

### Prognostic evaluation of the model

**Fig. 6:**
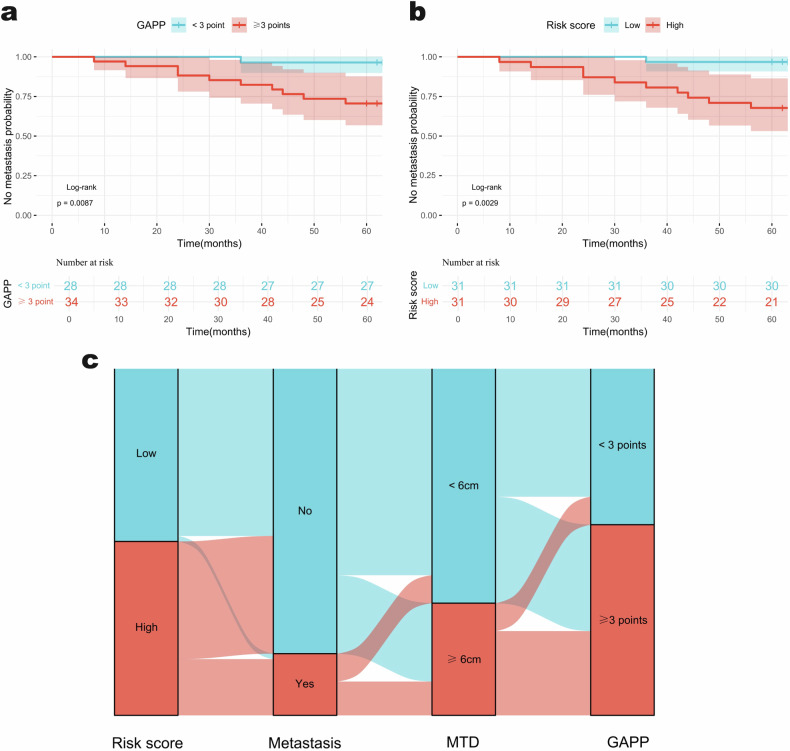
Predictive ability of GAPP (**a**) and the combined model’s risk scores (**b**) for metastasis potential in pheochromocytoma and paragangliomas, evaluated using Kaplan–Meier analysis based on MFS. **c** Sankey diagram illustrating the correspondence between risk score subgroups, metastasis status, MTD, and GAPP status

A total of 62 patients were successfully followed up in institutions A and C, with a median follow-up time of 75.5 months (range: 8–116 months). Among these patients, 11 experienced metastasis, resulting in a 5-year metastasis rate of 17.7%. The difference in MFS between patients with GAPP ≥ 3 points and those with GAPP < 3 points was statistically significant (HR, 9.421; 95% CI: 1.205–73.634; *p* = 0.009) (Fig. 6a). To evaluate the prognostic stratification of the combined model, patients were divided into low-risk and high-risk groups based on the median risk score. Kaplan–Meier survival analysis demonstrated that the combined model effectively stratifies MFS outcomes (HR, 11.647; 95% CI: 1.490–91.052; *p* = 0.003) (Fig. 6b). The Sankey diagram illustrates the distribution of patients within low- and high-risk groups, along with their metastasis rates, MTDs, and GAPP status (Fig. 6c).

## Discussion

PPGLs are rare neuroendocrine tumors, with 10–30% of cases potentially exhibiting metastatic characteristics [3, 8]. A large-scale retrospective study involving 2920 patients revealed that 320 of these patients exhibited metastasis. Notably, it was highlighted that 36.8% of patients with metastatic PPGL were misdiagnosed as benign at the time of initial diagnosis, and 59.6% of patients had metastatic lesions undetected during their initial surgery [14]. These findings underscore the challenges in identifying PPGLs with metastatic potential, especially in the absence of clear evidence of distant metastasis. While surgical resection remains the preferred treatment option, managing metastatic cases continues to be a significant challenge, thereby highlighting the critical need for early diagnosis and effective treatment. With advances in minimally invasive techniques, particularly the refinement of tumor dissection methods, tumor size is no longer the sole determining factor for surgical approach [34, 35]. Early evaluation of a tumor’s metastatic potential will become essential in selecting laparoscopic minimally invasive resection and impacting patient prognosis.

Previous studies have confirmed the feasibility of using radiomics to predict the metastatic potential of PPGLs [29]. indicating that the radiomic features captured during the venous phase demonstrate high stability and significant weight. With the advent of deep transfer learning techniques, pretrained DL networks can now be fine-tuned on smaller datasets, representing a new direction for advancements in radiomics research [25]. Therefore, to enhance clinical applicability by streamlining the acquisition of CT phases, this study utilized preoperative imaging during the venous phase, integrating radiomics and DL techniques to predict the metastatic potential of PPGLs. We selected six radiomic features, including maximum three-dimensional diameter shape features, first-order features, and texture features derived from wavelet decomposition. These features reflect tissue intensity, grayscale distribution, and heterogeneity, aligning with findings from previous research [36, 37]. Notably, we observed that three-dimensional image models were prone to overfitting during training, likely due to the limited sample size [38]. Consequently, we opted for a two-dimensional CNN model, which offers advantages such as a simpler network structure and shorter computation times, making it more suitable for practical clinical applications. We constructed a combined model that integrates intratumoral necrosis features, radiomics, and DL signatures. It demonstrates high accuracy in preoperatively predicting metastatic potential, assisting surgeons in identifying low-risk patients who may benefit from laparoscopic surgery. Conversely, high-risk patients are prioritized for open surgery to ensure complete tumor removal and reduce the risk of local recurrence and metastasis. Our research findings suggest that intratumoral necrosis in PPGLs may be a crucial indicator of tumor biological behavior. Extensive necrosis is often associated with rapid tumor growth or increased invasiveness, which may correlate with metastasis and poor prognosis [39].

Numerous previous studies have shown a significant correlation between tumor size and metastasis in PPGLs [3, 8], which aligns with our findings regarding shape features from radiomics. The endocrine guidelines recommend open surgery for pheochromocytomas larger than 6 cm or those with metastasis; however, they do not provide detailed criteria for high-risk factors. Consequently, physicians often rely on clinical judgment when determining surgical approaches for tumors at the 6-centimeter threshold. To this end, we explored the predictive performance of the combined model regarding the metastatic potential of pheochromocytomas measuring less than 6 cm. Our results demonstrate that the combined model reliably predicts the metastatic potential of tumors ≤ 6 cm, with validation from external datasets. Analysis of the model’s prognostic evaluation demonstrates that the risk score effectively stratifies postoperative metastasis risk. This aligns with previous studies, indicating a significant correlation between GAPP and the 5-year metastasis rate [14, 15, 18]. Compared to low-risk patients, those in the high-risk group typically require more frequent follow-up after surgery, including regular imaging studies and laboratory monitoring, to facilitate early detection and intervention of potential metastasis [40]. The Sankey diagram provides a visual representation that suggests patients with a diameter of ≤ 6 cm and a higher risk score may have an increased potential for metastasis, indicating that open resection could be a consideration for them. Conversely, patients with a low-risk score display a significantly lower likelihood of metastasis, making them more suitable candidates for minimally invasive surgical removal. In the case of tumors exceeding 6 cm in diameter, if their metastasis potential is assessed to be low, minimally invasive surgical removal may also warrant consideration. This illustrates the important value of the combined model in the early identification of patients with metastatic potential, thereby assisting these patients in benefiting from tailored surgical options and enhanced post-operative follow-up, ultimately aiming to improve overall prognosis.

This study has several limitations. First, the rarity of PPGLs constrained our sample size, limiting the differentiation between moderate/poorly differentiated and well-differentiated patients. Future research should aim to include all three differentiation types. Second, the combination of anatomic imaging and functional imaging offers additional value in enhancing specificity and sensitivity in diagnosis, as well as in its connection with targeted radionuclide therapy [7]. Future research incorporating Gallium-68-DOTATATE and Iodine-123-MIBG PET-CT could further improve the predictive performance of our analysis. Lastly, although we have integrated tumor necrosis characteristics, radiomics, and DL signatures to assess the metastatic potential of PPGL, artificial intelligence still faces numerous challenges in clinical applications. These challenges include issues related to data privacy and security, as well as the robustness and interpretability of algorithms [41]. Therefore, future clinical practices need to develop internal software to facilitate easier access to risk scores for every clinician.

## Conclusions

This combined model can predict the metastatic potential of PPGLs early in the preoperative phase. Additionally, it serves as a valuable clinical tool for preoperative risk assessment and surgical planning, particularly concerning the surgical threshold of 6 cm for pheochromocytomas.

## Supplementary information

ELECTRONIC SUPPLEMENTARY MATERIAL

## Data Availability

The datasets used and analyzed during the current study are available from the corresponding author upon reasonable request.

## Abbreviations

AUC: Area under the curve
CNN: Convolutional neural networks
CT: Computed tomography
D score: Deep learning signature score
DCA: Decision curve analysis
DL: Deep learning
ET: Extra trees
GAPP: Grading system for adrenal pheochromocytoma and paraganglioma
ICC: Intra/inter-class correlation coefficient
IVP: Intratumoral vascular penetration
KNN: K-nearest neighbors
LR: Logistic regression
MFS: Metastasis-free survival
MTD: Maximum tumor diameter
PPGL: Pheochromocytoma and paraganglioma
R score: Radiomics signature score
RF: Random forest
ROI: Region of interest
SVM: Support vector machine
XGBoost: EXtreme gradient boosting
