# Supplementary material for: CT-based radiomics deep learning signatures for non-invasive prediction of metastatic potential in pheochromocytoma and paraganglioma: a multicohort study
Source: Insights Imaging. 2025 Apr 5;16:81. doi: 10.1186/s13244-025-01952-4 (PMC11971077; doi:10.1186/s13244-025-01952-4)
Supplement: Supplementary file 1 — ELECTRONIC SUPPLEMENTARY MATERIAL [file 13244_2025_1952_MOESM1_ESM.pdf]

CT-Based Radiomics Deep Learning Signatures for Non-Invasive Prediction of Metastatic Potential in Pheochromocytoma and Paraganglioma:a multicohort study

ELECTRONIC SUPPLEMENTARY MATERIAL

1. CT Scan methodology

This study utilized different CT scanners from three hospitals, including a 256-channel CT scanner (Siemens), three 128-channel CT scanners (two from Siemens and one from General Electric), two 256-channel CT scanners (both from Philips), and a 320-channel CT scanner (Toshiba). All patients underwent routine non-enhanced scans and contrast-enhanced abdominal CT scans. Detailed information regarding the scanning and reconstruction settings for the seven CT scanners can be found in Table S1. After the non-contrast CT scan, a mechanical injector administered iodinated contrast agent (300 mg I/mL) through the elbow vein at a flow rate of 2.5-3.0 mL/s. Images for the arterial and venous phases were acquired at 30 seconds and 60 seconds post-injection, respectively.

Table S1 CT scanning protocol for patients with PPGLs

Manufacture	SIEMENS	SIEMENS	SIEMENS	Philips	Philips	Toshiba	General Electric
CT scanner	CT256	CT128	CT128	CT 256	CT256	CT320	CT128
Scanner mode	SOMATOM Drive	SOMATOM Defifinition AS	SOMATOM Defifinition Edge	Brilliance iCT	IQon Spectral CT	Aquilion ONE	Revolution Fro
Tube voltage (kV)	120	120	120	120	120	120	120
Tube current (mA	250	250	200	300-350	300	200	Automas,300-350
Rotation time (s)	0.5	0.5	0.5	0.5	0.4	0.5	0.8
Collimation (mm)	64*0.6	64*0.6	64*0.6	128*0.625	64*0.625	160*0.625	64*0.625
Slice thickness (mm)	1.25	2	1	2	1	3	1
Matrix	512 × 512	512 × 512	512 × 512	512 × 512	512 × 512	512 × 512	512 × 512
Field of view (mm)	350	350	350	350	350	350	350
Kernel	standard	standard	B30f	Stardand	standard	Fc10	standard

CT,Computerized Tomography; PPGLs, Pheochromocytoma/Paragangliomas

2. Definitions of some radiological features [1]

A circular region of interest is placed in the tumor to measure attenuation values. These values are measured by two radiologists and the average is taken as the final result:

(a) Tumor location: adrenal (paraganglioma), or extra-adrenal (paraganglioma);

(b) MTD: Maximum tumor diameter;

(c) Presence of calcification, with CT attenuation value > 100 HU on non-enhanced CT scan;

(d) Presence of necrosis changes when the CT attenuation value on non-enhanced CT scan is <20 HU, and the enhancement amplitudes in both arterial and venous phases are <10 HU;

(e) Intratumoral hemorrhage refers to a CT attenuation value between 55 and 90 on non-enhanced CT scan;

(f) The unclear margin of a tumor refer to the increased density of surrounding fat or the blurred demarcation between the tumor and adjacent tissues.

(g) IVP:the observation of intratumoral vascular penetration was made on the CT-enhanced images.

3. Surgical Histopathologic Analyses

The patients recruited from the three institutions underwent tumor resection and immunohisto-chemical analysis to ensure complete tissue sampling for pathological examination. Tissue specimens were serially sectioned into 3-4mm slices and stained with hematoxylin and eosin. To ensure accurate pathological evaluation, two careful examinations were conducted - initially and at the first follow-up visit 1 month later. This method aims to minimize the possibility of measurement errors. The detailed GAPP scoring criteria are shown in Table S2

Table S2 The GAPP grading system for patients with PPGLs

parameters	Points scored
Histological pattern	
Zellballen	0
Large and irregular cell nest	1
Pseudorosette (even focal)	1
Cellularity	
Low (<150 cells/U)	0
Moderate (150–250 cells/U)	1
High (>250 cells/U)	2
Comedo-type necrosis	
Absence	0
Presence	2
Vascular or capsular invasion	
Absence	0
Presence	1
Ki67 labeling index (%)	
<1	0
1–3	1
>3	2
Catecholamine type	
Non-functioning	0
Adrenergic type	0
Noradrenergic type	1
Total maximum score	

If the urine fractionated metanephrine (UMN) levels were high with or without elevated urine fractionated normetanephrine (UNM) levels,the catecholamine type was adrenergic type.If the UNM levels were high without elevated UMN levels, the catecholamine type was noradrenergic type.

Table S3. Inter-observer Consistency in Pathological Assessment.

Pathological Assessment	Disagreement	Kappa value	95% CI
GAPP	16(6.4%)	0.921	0.884, 0.956
Low/high-risk group	1(0.4%)	0.992	0.973, 1.000

4. Parameter Settings for the CNN Model

We employed convolutional neural network models, specifically ResNet18, ResNet34, ResNet50, and ResNet101, which were pre-trained on the ImageNet dataset for transfer learning[2]. The training process was conducted on the "Onekey AI" platform (http://www.medai.icu/) using PyTorch version 1.8.0.

Parameters for 2D Maximum Cross-Section Images: To classify patients with PPGL into low-risk and high-risk groups, we modified the fully connected layer's output from 1000 to 2. The training data were derived from images highlighting the largest areas of interest. Grayscale values of the images were normalized to a range of [-1, 1] employing min-max scaling. Each cropped sub-region image was resized to 224×224 pixels using nearest-neighbor interpolation, ensuring consistency with the network’s input size. We utilized a cross-entropy loss function to update the network weights and train the model for the prediction task. An adaptive learning rate optimizer with an initial learning rate of 0.01 was utilized, training for 50 epochs with a batch size of 32.

Parameters for 3D Images Capturing the Entire Tumor: The training dataset consisted of 3D images that captured the entire tumor, pre-outlined using ITK-SNAP software (version 3.6.0, http://www.itksnap.org). The grayscale values of these images were normalized to a range of [-1, 1] and resized to a uniform dimension of 96×96×96 mm^3, allowing them to serve as input for the model. This size ensured that all tumors within the volumes of interest, along with their surrounding environments, were effectively incorporated into the deep learning model for thorough analysis. The model's hyperparameters included a batch size of 4 and an initial learning rate of 0.001, with training conducted over 50 epochs. The optimization process utilized the Adam optimizer, and the loss function was defined as the cross-entropy function, suitable for classification tasks.

During the training process, these parameters were frozen to prevent further updates, allowing only the final linear classification layer to be trained. Once the loss on the training set stabilized, the parameters of the feature extraction component were unfrozen to permit additional updates, followed by further training on the training set. After the loss stabilized again, the weights of the model at that stage were saved as the optimal model. This strategy, commonly adopted in transfer learning, effectively reduces the risk of overfitting. Upon completion of the model training, the testing dataset was introduced into the deep learning model to obtain predicted probabilities for the target classes.

5. Statistical Analysis

Statistical analyses were conducted using R software (version 4.2.3; https://www.r-project.org) and Python software (version 3.5.6; http://www.python.org). Continuous variables were compared using the t-test (for normally distributed data) or the Mann-Whitney U test (for non-normally distributed data), while categorical variables were analyzed using the chi-squared test. The 95% confidence intervals for the area under the curve were determined using a bootstrap method with 1,000 replications. The generation of petal plots, radar charts, and Sankey diagrams was accomplished using the "ggplot2" package, while heat maps were created using the "pheatmap" package. The "pROC" package facilitated the construction of ROC curves. Univariate and multivariate logistic regression analyses, as well as Kaplan-Meier curve analysis and calibration curve creation, were performed with the "rms", "survminer", and "survival" packages. Calibration curves were derived using bootstrapping with 1,000 resamples, and the models were evaluated using the Hosmer-Lemeshow goodness-of-fit test. Clinical decision curves were plotted using the "dcurves" package. A two-tailed p-value < 0.05 indicates significant difference.

6. The results of feature selection based on LASSO

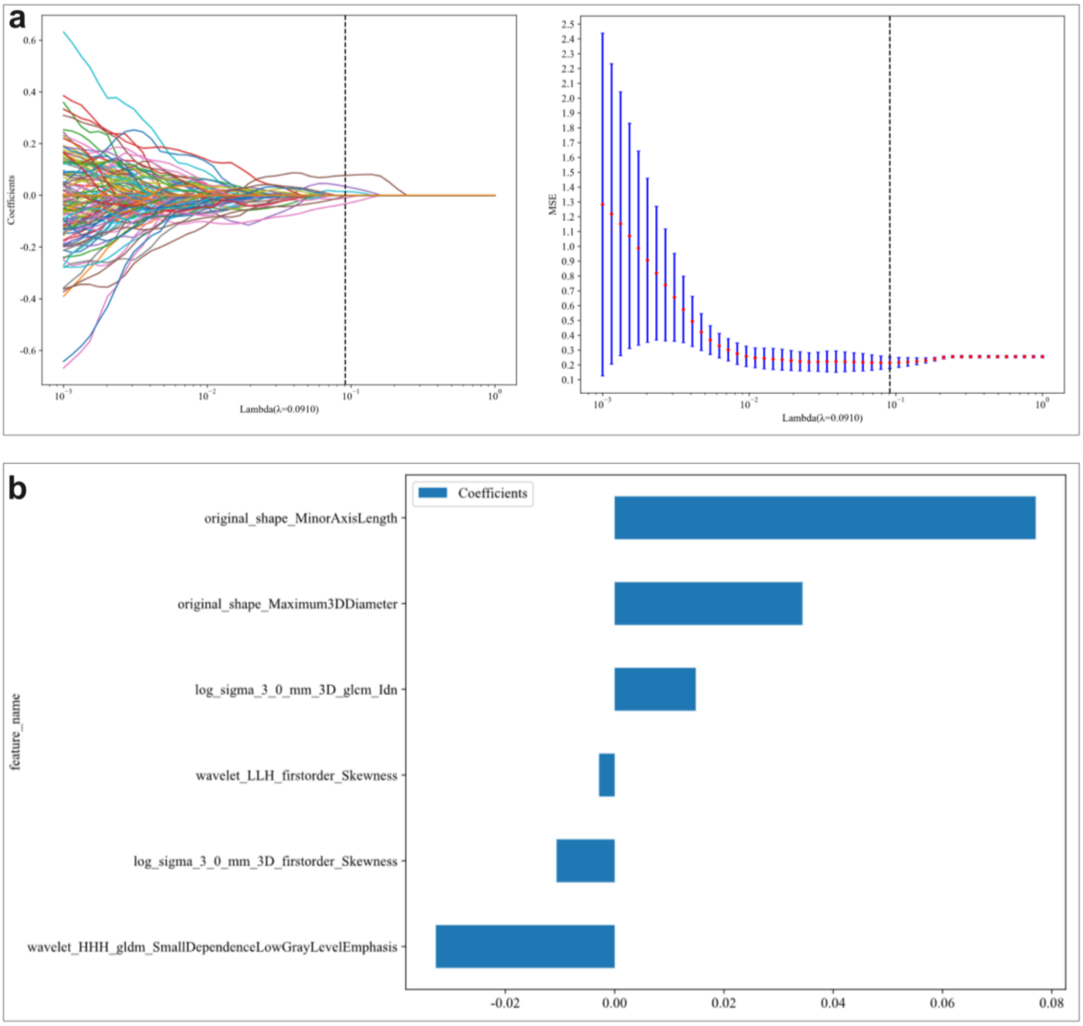

Figure S1. Radiomic feature selection was performed using LASSO regression, with 10-fold cross-validation based on the minimum criterion. (b) A histogram of feature weights based on LASSO coefficients is presented. LASSO, least absolute shrinkage and selection operator.

7. Visualization of deep learning models.

To investigate the interpretability of the deep learning models and visualize their outputs, this study employed Gradient-weighted Class Activation Mapping (Grad-CAM). Grad-CAM utilizes the output feature maps from the last convolutional layer for activation, generating rough local feature localization maps that highlight the regions in the images that are significant for predicting the target classification. Figure S2 presents examples of Grad-CAM outputs generated from 2D maximum cross-section images trained using the ResNet model.

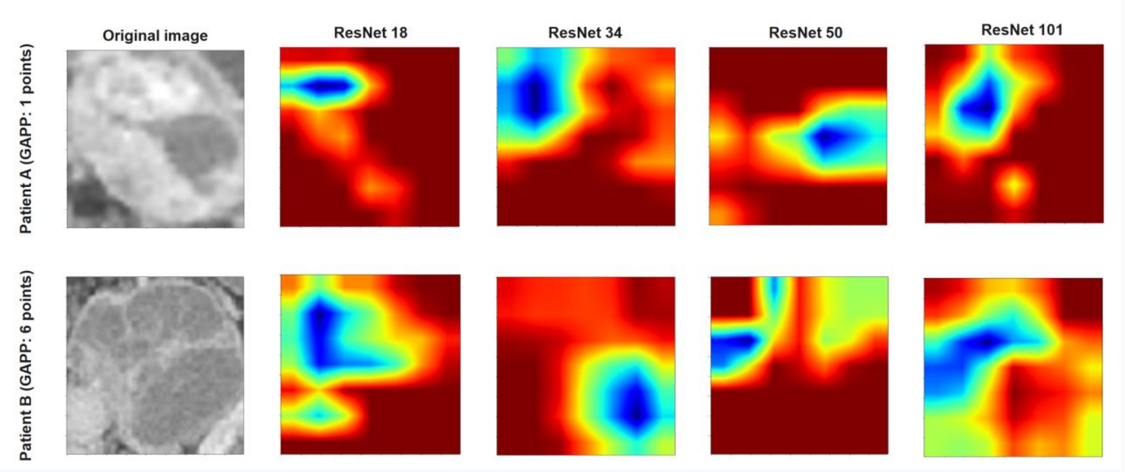

Figure S2. Comparison of Grad-CAM from different deep learning models in low-risk PPGL patient A and high-risk patient B.

References

1. Niu, Z., Wang, J., Yang, Y., He, J., Wang, S., Xie, Z., Shao, M., & Zhu, F. (2022). Risk prediction model establishment with tri-phasic CT image features for differential diagnosis of adrenal pheochromocytomas and lipid-poor adenomas: Grouping method. Frontiers in endocrinology, 13, 925577. https://doi.org/10.3389/fendo.2022.925577

2．He K, Zhang X, Ren S, Sun, J. Deep residual learning for image recognition (2016) Proceedings of the IEEE Conference on Computer Vision and Pattern Recognition 770-778
